# Childhood maltreatment, shame, psychological distress, and binge eating: testing a serial mediational model

**DOI:** 10.1186/s40337-023-00819-7

**Published:** 2023-06-13

**Authors:** Elyse O’Loghlen, Roslyn Galligan, Sharon Grant

**Affiliations:** Department of Psychological Sciences, Swinburne University of Technology, Hawthorn, VIC 3122 Australia

**Keywords:** Binge eating disorder, Feeding and eating disorders, Eating disorders, Shame, Depression, Anxiety, Stress

## Abstract

**Objective:**

Despite evidence of causal relationships between childhood maltreatment and the development of binge eating disorder (BED), research on mediating mechanisms is lacking. The present study sought to understand the childhood maltreatment-binge eating relationship more fully by examining three types of shame (internal, external, body) and psychological distress as mediators in this relationship. There is evidence that shame and psychological distress are associated with both childhood maltreatment and binge eating pathology. It was hypothesised that shame stemming from childhood maltreatment would contribute to psychological distress, and to binge eating as a dysfunctional emotion regulation strategy, in a serial mediational model.

**Method:**

Five hundred and thirty adults with self-reported binge eating symptoms completed an online survey, which included measures of childhood maltreatment, internal shame, external shame, body shame, psychological distress, and binge eating and other eating disorder symptoms.

**Results:**

Path analyses showed three specific relationships: (1) a relationship between childhood emotional maltreatment and binge eating, which was serially mediated by internal shame and psychological distress; (2) a relationship between childhood sexual abuse and binge eating, which was mediated by body shame; and (3) a relationship between childhood physical maltreatment and binge eating, which was mediated by psychological distress. We also found a feedback loop, whereby binge eating might lead to increased overvaluation of body shape and weight (possibly due to increased weight) and then to an increase in internal shame and body shame. The final model showed excellent fit for the data.

**Discussion:**

Findings extend our understanding of the link between childhood maltreatment and BED. Future intervention research should focus on examining the efficacy of interventions for different forms of childhood maltreatment, based on the key mediating factors.

## Abstract (plain language summary)

Despite research showing a clear link between past childhood maltreatment (e.g., childhood emotional abuse, sexual abuse, physical abuse, neglect) and developing binge eating disorder as an adult, research cannot currently fully explain why this link exists. In this study, we explored several ways that childhood maltreatment might lead to binge eating. We examined whether different types of childhood maltreatment led to increased levels of shame or psychological distress (e.g., symptoms of depression, anxiety, and stress), and whether increased shame or psychological distress then contributed to the use of binge eating as a strategy to manage these emotions (e.g., through emotional binge eating). The results of our study showed that specific types of childhood maltreatment did lead to increased shame and binge eating. For instance, we found that adults who had experienced childhood emotional abuse tended to experience higher levels of shame about themselves, as well as higher levels of psychological distress and higher levels of binge eating. Findings from this study suggest that specific types of therapy (e.g., compassion-focused therapy for eating disorders for high levels of shame in eating disorders) might be useful for adults who have experienced childhood maltreatment and experience high levels of shame and psychological distress that accompany their binge eating.

## Introduction

Research consistently shows a link between childhood maltreatment and the development of eating disorders (EDs) [10]. Prevalence rates of childhood maltreatment appear to be approximately two to four-fold higher in the ED population compared to the general population, with adults with EDs who have experienced childhood maltreatment reporting earlier age of ED onset and more severe illness presentation [50]. Across ED type, bulimia nervosa (BN) and binge eating disorder (BED) show the highest rates of childhood maltreatment [10, 50]. Within BED, estimates of child maltreatment range from 52 to 83%. The most frequently reported types of childhood maltreatment within BED are childhood emotional neglect (CEN; 49–69%), childhood emotional abuse (CEA; 46–59%), childhood physical neglect (CPN; 32–50%), childhood physical abuse (CPA; 28–36%), and childhood sexual abuse (CSA; 26–32%), respectively [1, 5, 33]. Limited past research in this area has found links between CEA and binge eating via self-criticism [25] and anger [24], as well as from CEA and CPN to binge eating via alexithymia [49]. However, mediating mechanisms in the childhood maltreatment-BED relationship across all five forms of childhood maltreatment have not been thoroughly explored [59].

The link between childhood maltreatment and the development of psychological disorders in adulthood is well documented [38]. For example, among adults with BED, links have been found between CEA and dysthymic disorder, from CSA and CPN to post-traumatic stress disorder, and between CPA and alcohol use disorder [5]. These aetiological pathways suggest that childhood maltreatment contributes to increased psychological distress which persists into adulthood. The link between psychological distress and the development of binge eating and BED is also well-established [68], explained broadly by the use of binge eating as a dysfunctional strategy to regulate mood [53, 55]. However, it remains unclear how specific emotional or self-evaluative processes might mediate the relationship between childhood maltreatment and psychological distress in the BED population.

A presently unexplored factor which might explain the link between childhood maltreatment and psychological distress is shame. The experience of chronic shame is a known consequence of childhood maltreatment [15], and is also linked to the development and maintenance of BED [57]. Shame can be defined as a self-evaluative emotion characterised by global, negative self-evaluations, or the perception of having one’s flaws exposed [64]. Shame can be further categorised as either internally focussed, the experience of negative self-judgement and feelings of inadequacy and inferiority (‘internal shame’) or externally focussed, the perception of negative evaluations from others about the self (‘external shame’) [27]. ‘Body shame’ describes feelings of inadequacy focussed specifically on one’s appearance and body, and is also predicted by childhood maltreatment [6].

Both internal shame and external shame are linked to childhood maltreatment [45, 66]. Specifically, CEA and CEN have been linked to the development of internal shame [61, 66]. The relationship between childhood maltreatment and external shame is less clear, with several studies finding a relationship between childhood adverse experiences such as peer victimisation, as well as experiences of criticism and rejection by parents to external shame [18, 74]. Furthermore, two of these studies [61, 74] found that internal and external shame then predicted depressive symptoms which are indicative of psychological distress [17].

Several studies have found that CSA is linked to the development of body shame [3, 48, 69] and internal shame [71]. Furthermore, the review by Whiffen and MacIntosh [71] found that internal shame and body shame were key mediators in the relationship between CSA and emotional distress. These findings suggest that there are a range of pathways from childhood maltreatment to the development of psychological distress: from CEA, CEN, and CSA via internal shame, from childhood maltreatment via external shame, and from CSA via body shame.

Internal shame, external shame, and body shame have also been linked to the development of BED [57]. There is a general lack of research examining early experiences as a predictor of shame in the BED population, however recent research indicates that early shame-inducing memories [18] and experiences of body shaming [20] play a role in the development of shame in the BED population.

Considering the links between childhood maltreatment and psychological distress via the three types of shame, as well as the link between psychological distress and the development of BED [68], it is possible that the relationship between childhood maltreatment and the development of BED may be serially mediated by shame and psychological distress. A better understanding of the mechanisms mediating the childhood maltreatment-BED relationship is critical for the development of targeted psychological treatment for the significant proportion of individuals with BED with a history of childhood maltreatment.

### The current study

The primary aim of this study was to examine mediating factors in the relationship between childhood maltreatment and binge eating pathology. Specifically, the study examined whether three types of shame (internal, external, body) and psychological distress independently or serially mediated the link between five types of childhood maltreatment (CEA, CPA, CSA, CEN, CPN) and binge eating pathology. We expected different types of shame stemming from childhood maltreatment would contribute to psychological distress and then binge eating as a coping strategy. Considering previous research, we hypothesised that:

1. The relationship between CEA, CEN, and CSA to binge eating would be serially mediated by internal shame and psychological distress;
2. The relationship between childhood maltreatment and binge eating would be serially mediated by external shame and psychological distress; and
3. The relationships between CSA to binge eating would be serially mediated by body shame and psychological distress.

## Method

### Participants and procedure

Adults (≥ 18 years old) with self-reported binge eating symptoms were eligible for study participation. Participants were recruited from July 2021 to January 2022 through an online advertisement linked to an online survey, which was placed on ED, BED, and overeating support forums and social media platforms. Consent was obtained prior to beginning the survey. Participants were invited to enter a draw to receive a gift card valued at $100AUD at the completion of the survey. All procedures were approved by an institutional Human Research Ethics Committee (project 20215477-8486). Participants with a score below 17 on the Binge Eating Scale (*n* = 58), indicating non-pathological levels of binge eating [28], were removed from analysis. The total sample (*N* = 530) was predominantly female, White, residing in a western country, holding a bachelor’s degree or other tertiary qualification, and at the low end of the ‘obese’ body mass index (BMI) category [73]. Table 1 shows detailed participant demographics.

**Table 1:** Participant demographics

	*N* = 530	%
Gender		
Female	408	76.70
Male	70	13.21
Non-binary	48	9.06
Prefer not to say	4	0.75
Highest level of education completed		
Secondary school	186	35.09
Bachelor’s degree	183	34.53
Postgraduate degree	72	13.58
Certificate/diploma	42	7.92
Some school	33	6.23
Doctorate	14	2.64
Ethnicity		
White	403	76.04
Asian	45	8.49
Multiple	34	6.42
Other/prefer not to say/do not know	29	5.47
Black	10	1.89
Hispanic	9	1.70
Country of residence		
United States of America	262	49.43
Australia	86	16.23
Other/prefer not to say	78	14.72
United Kingdom	43	8.11
Canada	36	6.79
Germany	15	2.83
New Zealand	10	1.89
Childhood maltreatment		
Childhood emotional neglect	347	65.47
Childhood emotional abuse	310	58.49
Childhood physical abuse	209	39.43
Childhood sexual abuse	174	32.83
Childhood physical neglect	97	18.30

	*M*	*SD*
Age	28.35	9.47
BMI ranges (kg/m^2^)	30.41	11.87
18.5–24.9	194	36.60
25–29.9	97	18.30
≥ 40	95	17.93
30–34.9	64	12.08
Obese class II (35–39.9)	45	8.49
< 18.5	35	6.60

The sample was further categorised into two subgroups according to whether or not individuals met probable criteria for bulimia nervosa (BN) based on the Diagnostic and Statistical Manual of Mental Disorders, Fifth Edition (DSM-5) criteria [2] using the Eating Disorder Examination-Questionnaire (EDE-Q). Probable BN was defined as (a) ≥ four episodes of objective binge eating accompanied by a sense of loss of control, and (b) ≥ four episodes of purging (vomiting and/or laxative use), and/or (c) ≥ 13 episodes of fasting (8 waking hours or more), and/or (d) ≥ 20 episodes of excessive exercise in the last 28 days [42, 51]. According to these criteria, a subset of the total sample (*n* = 167; 31.5%) met criteria for BN.

### Survey measures

#### Demographic information

Demographic questions included sex, age, height and weight, ethnicity, country of residence, and highest level of education completed.

#### Adverse childhood experiences-questionnaire (ACE-Q)

The ACE-Q [26] is a 10-item dichotomous (yes/no) measure of 10 types of adverse childhood experiences (ACEs) that have occurred in childhood (< 18 years old), five involving child maltreatment (emotional abuse [CEA], physical abuse [CPA], sexual abuse [CSA], emotional neglect [CEN], or physical neglect [CPN]). These five types of childhood maltreatment were examined due to their high prevalence in the BED population [33, 50]. The ACE-Q shows strong test–retest reliability [22] and concurrent validity [54, 65]. In the present sample, Cronbach’s alpha was 0.67.

#### Internalised shame scale–shame subscale (ISS-S)

The ISS-S [12] is the 24-item subscale of the internalised shame scale (ISS) which measures internal shame. Items are rated on a scale from 0 (never) to 4 (almost always). Items are summed to provide a global score (0–96), with higher scores indicating a greater degree of internal shame. The ISS-S shows good internal consistency and concurrent validity [16]. In the present sample, Cronbach’s alpha was 0.95.

#### Other as shamer scale-2 (OAS-2)

The OAS-2 [44] is an 8-item short form version of the other as shamer scale which measures external shame. Items are rated on a scale from 0 (never) to 4 (almost always). Items are summed to provide a global score (0–32), with higher scores indicating a greater degree of external shame. The OAS-2 shows good internal consistency and concurrent validity [44]. In the present sample, Cronbach’s alpha was 0.92.

#### Body image shame scale (BISS)

The BISS [19, 21] is a 14-item measure of body shame. Items are rated on a scale from 0 (never) to 4 (almost always). Items are summed to provide a global score (0–56), with higher scores indicating a greater degree of body shame. The BISS shows very good internal consistency and concurrent validity [19, 21]. In the present sample, Cronbach’s alpha was 0.91.

#### Depression anxiety stress scale-21 (DASS-21)

The DASS-21 [41] is a 21-item measure of depression, anxiety, and stress symptoms. Items are rated on a scale from 0 (never) to 3 (almost always). Items are summed and multiplied by two to provide a global score (0–126), with higher scores indicating a greater degree of psychological distress. The DASS-21 shows good internal consistency and concurrent validity [56]. In the present sample, Cronbach’s alpha was 0.92.

#### Binge eating scale (BES)

The BES [30] is a 16-item measure of binge eating symptoms. Items comprise three to four response options, which correspond to a level of severity on a range from 0 to 3. Items are summed to provide a global score (0–46), with higher scores indicating a greater degree of binge eating. Global scores can be categorised into subclinical/minimal binge eating (≤ 17), moderate binge eating (18–26), and severe binge eating (≥ 27). The BES shows good internal consistency and concurrent validity [11], and is considered a valid screening tool for clinically significant binge eating (score ≥ 18) [19, 21]. In the present sample, Cronbach’s alpha was 0.80.

#### Eating disorder examination-questionnaire (EDE-Q)

The EDE-Q [23] is a 28-item measure of eating disorder cognitions and behaviours. Twenty-two items are rated on a scale from 0 (no days) to 6 (every day) and subscale items are summed and divided by number of items to provide four subscale scores (0–6): restrained eating, eating concern, weight concern, and shape concern. To measure overvaluation of shape and weight, the subscales weight concern and shape concern were summed and divided by two. Six items measure the frequency of eating disorder behaviours (e.g., episodes of loss-of-control binge eating, vomiting, laxative use, overexercise) experienced in the last 28 days, and which were used in the present study to classify probable ED diagnoses. The EDE-Q is considered the gold standard as a questionnaire-based assessment of eating disorder symptoms and probable eating disorder diagnosis [52].

## Results

### Descriptive statistics

There was no missing data. Descriptive and correlational analysis were conducted using IBM SPSS software (version 28). Multicollinearity was assessed using the variance inflation factor (VIF); VIF values above five were considered problematic [14]. As all independent variables had a VIF below five, collinearity was considered acceptable.

Analysis of variance (ANOVA) calculations were used to assess gender differences across all measures. No significant differences were found across gender according to binge eating symptoms (*F*[2, 527] = 2.01, *p* = 0.14) or body shame (*F*[2, 527] = 1.32, *p* = 0.27). Tukey’s post-hoc tests showed significant (*p* < 0.05) gender differences across psychological distress (*F*[2, 527] = 8.23, *p* < 0.001), external shame (*F*[2, 527] = 4.86, *p* = 0.008) and internal shame (*F*[2, 527] = 6.22, *p* = 0.002), with non-binary individuals experiencing higher levels of psychological distress, external shame and internal shame than female or male individuals. While these findings are consistent with research indicating that non-binary individuals experience higher rates of depression and shame than those who identify with their sex assigned at birth [60, 63], they were not considered problematic for analysis due to the small size of this subgroup (9.06%) in the total sample. As expected (Table 2), all variables showed significant positive correlations. The four potential mediating factors (internal shame, external shame, body shame, psychological distress) were all significantly associated with childhood maltreatment and binge eating pathology, indicating that they should be considered as mediating variables.

**Table 2:** Means, standard deviations, and pearson correlations between study variables (N = 530)

	M (*SD*)	CPA	CEA	CSA	CPN	CEN	Internal shame	External shame	Body shame	Psychological distress	Binge eating
CPA	0.39 (0.49)	–									
CEA	0.58 (0.49)	0.52	–								
CSA	0.33 (0.47)	0.24	0.17	–							
CPN	0.18 (0.39)	0.33	0.26	0.20	–						
CEN	0.65 (0.48)	0.33	0.39	0.22	0.20	–					
Internal shame	71.26 (18.49)	0.13	0.28	0.12	0.09*	0.30	–				
External shame	21.99 (7.18)	0.12	0.25	0.12	0.15	0.31	0.82	–			
Body shame	41 (11.01)	0.15	0.20	0.16	0.12	0.18	0.59	0.56	–		
Psychological distress	64.97 (25.78)	0.21	0.29	*0.*12	0.19	0.28	0.68	0.58	0.42	–	
Binge eating	31.18	0.12	0.18	0.18	0.18	0.13	0.43	0.35	0.47	0.44	–
Overvaluation of shape and weight		0.11*	0.20	0.14	0.03^	0.16	0.51	0.42	0.59	0.35	0.46

All correlations significant at the 0.01 level (2-tailed) except those marked * which indicates significance at the 0.05 level, and that marked ^ which indicates *p* > 0.05

*CPA* childhood physical abuse, *CEA* childhood emotional abuse, *CSA* childhood sexual abuse, *CPN* childhood physical neglect, *CEN* childhood emotional neglect

### Path analyses

Path analyses were performed with IBM SPSS Amos software (version 26) using the maximum-likelihood estimation method. In line with the recommendations of Browne and Cudeck [7] and Hu and Bentler [37], the following fit indices were used to evaluate acceptable fit: comparative fit index (CFI) ≥ 0.95, standardized root-mean-square residual (SRMR) ≤ 0.08, and the root-mean-square error of approximation (RMSEA) ≤ 0.08. The resampling method bootstrap (with 1000 resamples) was used to create 95% bias-corrected confidence intervals to estimate direct and indirect effects.

A fully saturated mediated model was initially tested. This model did not show an acceptable fit with the data, χ^2^ (11, 530) = 1051.52, *p* < 0.001; CFI = 0.43; SRMR = 0.18; RMSEA = 0.42. Model modification indices suggested the addition of two paths, which are supported by current theory and/or past literature: (1) internal shame is a robust predictor of psychological distress [9, 13, 58], and (2) internal shame is also closely associated with body shame [29, 57]. The addition of a path from internal shame to psychological distress significantly improved model fit, ΔΧ^2^ (1) = 285.08, *p* < 0.001, as did the addition of a path from internal shame to body shame, ΔΧ^2^ (1) = 201.78, *p* < 0.001. The addition of these two paths led to overall acceptable model fit, Χ^2^ (8, 530) = 22.71, *p* = 0.004; CFI = 0.99; SRMR = 0.02; RMSEA = 0.06. Fitting direct paths from the five types of childhood maltreatment to binge eating symptoms did not significantly improve model fit, indicating that the relationships between childhood maltreatment experiences and binge eating symptoms were fully mediated. In partial support of hypothesis 1, internal shame and psychological distress serially mediated the relationship between childhood emotional maltreatment but and binge eating symptoms. Hypothesis 2 was also partially supported the relationship between some forms of child maltreatment (physical abuse, physical neglect) and binge eating symptoms was mediated by psychological distress but was not mediated by external shame. To test our third hypothesis, a path was fitted from body shame to psychological distress, however this path was not significant (*p* = 0.78) so it was removed from the model.

**Fig. 1:**
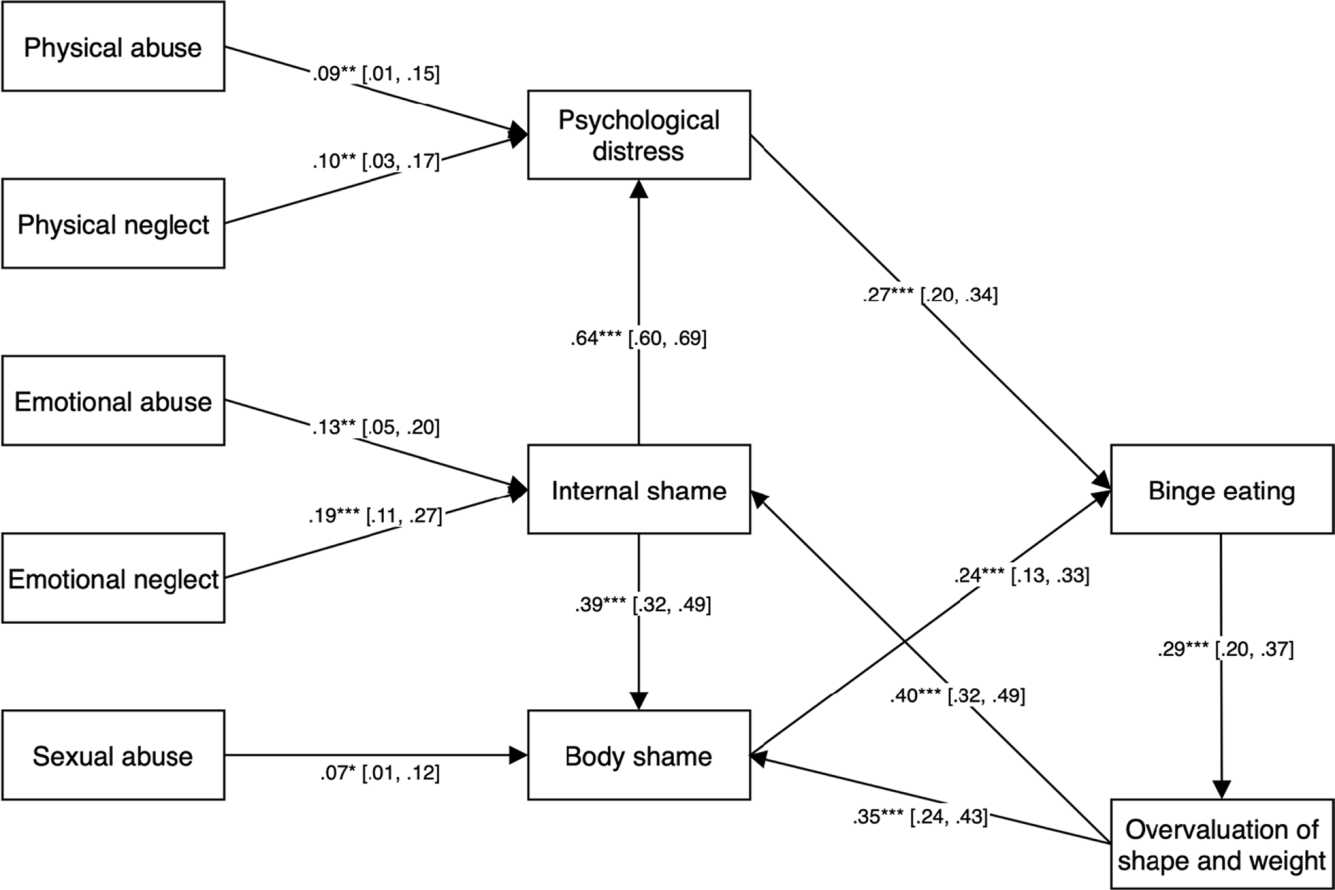
Final path model (N = 530).*Note*. Results are standardised path coefficients. Values in brackets are 95% confidence intervals. *p < 0.05, **p < 0.01, **p < 0.001

Non-significant regression coefficients were then trimmed from the model, including the removal of a direct path from internal shame to binge eating symptoms. As there were no significant direct or indirect regression paths leading from external shame to binge eating symptoms, this variable was removed from the model. Finally, based on sample BMI (more than two thirds BMI > 25) we tested a feedback loop whereby binge eating might lead to increased overvaluation of body shape and weight due to increased weight [34], and then to an increase in internal shame, body shame, and psychological distress [57]. The variable, overvaluation of shape and weight, and these four feedback paths were added to the model. Several significant paths were found and retained, including from binge eating to overvaluation of shape and weight, as well as from overvaluation of shape and weight to internal shame and body shame. The final model (Fig. 1) showed excellent fit, χ^2^ (22, 530) = 46.93, *p* = 0.002; CFI = 0.98 SRMR = 0.05; RMSEA = 0.04. This model accounted for 18%, 29%, 45%, 48%, and 27% of the variance in overvaluation of shape and weight, internal shame, body shame, psychological distress, and binge eating, respectively.

**Fig. 2:**
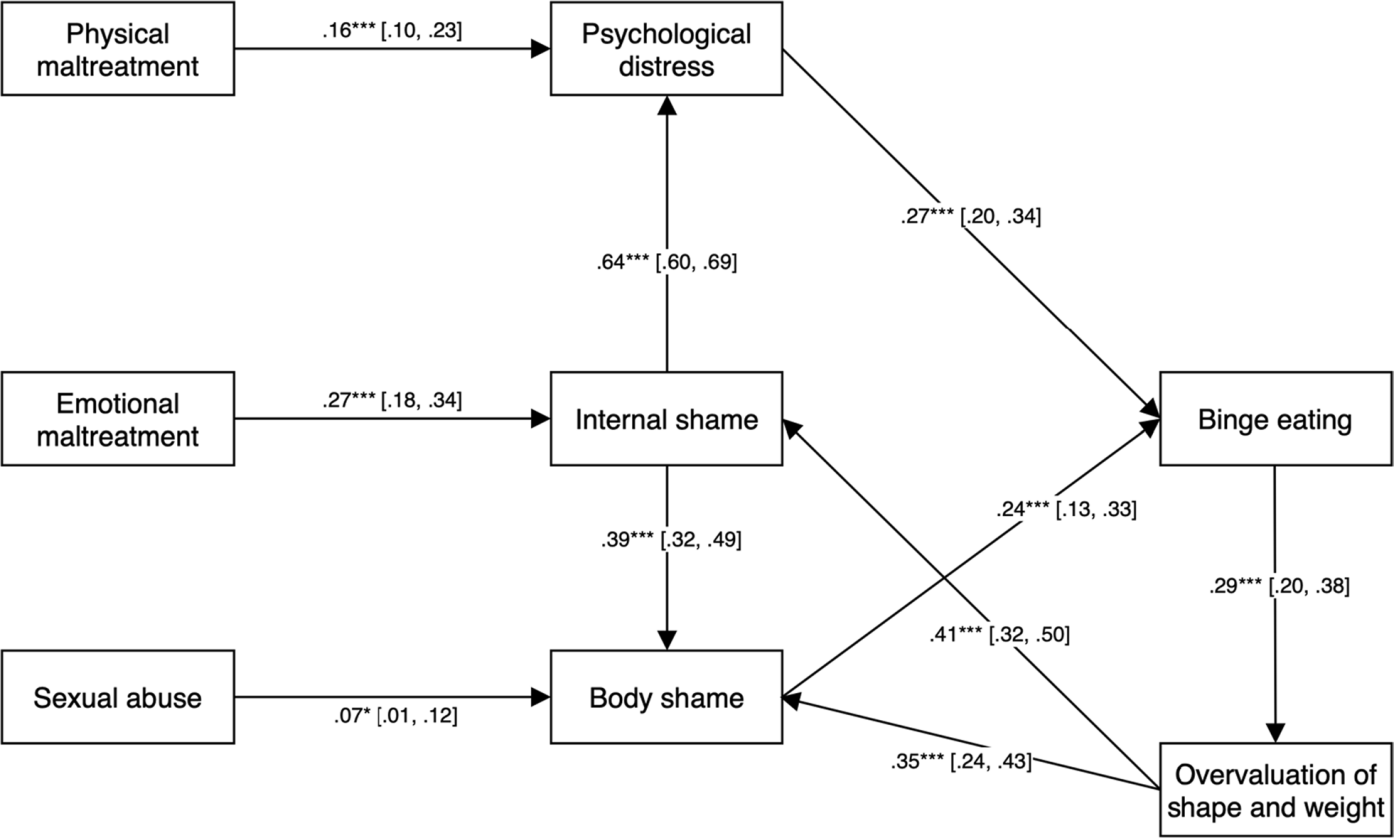
Final path model combining types of emotional and physical maltreatment (N = 530). *Note*. Results are standardised path coefficients. Values in brackets are 95% confidence intervals. *p < 0.05. ***p < 0.001)

Due to the significant proportion of participants with a probable diagnosis of BN, the final model was run again, stratified by eating disorder subsample (BED *n* = 363; BN *n* = 167). The final model was an excellent fit for the BED subsample, χ^2^ (23, 363) = 32.94, *p* = 0.082; CFI = 1.00 SRMR = 0.06; RMSEA = 0.04, and a good fit for the BN subsample, χ^2^ (23, 167) = 34.98, *p* = 0.052; CFI = 0.97, SRMR = 0.05; RMSEA = 0.06, indicating that the inclusion of the BN subsample did not significantly skew model fit. For parsimony, the final model was rerun, combining CEA with CEN into a total score to form an emotional maltreatment variable, and combining CPA and CPN into a total score to form a physical maltreatment variable. This model (Fig. 2) showed an excellent fit for the data, χ^2^ (11, 530) = 17.82, *p* = 0.09; CFI = 0.99, SRMR = 0.03; RMSEA = 0.03.

## Discussion

This study was the first of its kind to examine whether internal shame, external shame, body shame, and psychological distress independently or serially mediate relationships between different forms of childhood maltreatment and binge eating symptoms among adults. Our first hypothesis was partially supported: the relationships from childhood emotional maltreatment but not CSA to binge eating symptoms were serially mediated by internal shame and psychological distress. Our second hypothesis was partially supported: relationships between some childhood maltreatment and binge eating symptoms were mediated by psychological distress but were not mediated by external shame. Our third hypothesis was also partially supported; the relationship between CSA and binge eating symptoms was mediated by body shame but not psychological distress. Our results also suggested that the relationship between childhood physical maltreatment and binge eating is mediated by psychological distress. Finally, the feedback loop we tested showed that binge eating led to increased overvaluation of shape and weight, contributing to further increased internal shame and body shame.

Despite past research linking external shame to childhood maltreatment and binge eating [45, 57], our results showed that external shame did not mediate the childhood maltreatment-binge eating relationship for any of the five forms of childhood maltreatment examined in the study. Notably, internal shame and external shame were highly correlated, and childhood maltreatment and binge eating were more highly correlated with internal shame than external shame. Therefore, it is possible that there was no unique contribution of external shame to the model after accounting for internal shame [14]. Previous studies which found that external shame made a significant contribution to binge eating did not examine internal shame in their models [18, 20], providing support for this explanation. Additionally, our result was consistent with previous research examining internal shame and external shame together [47], whereby the link between internal shame and binge eating was significantly stronger than that between external shame and binge eating.

### Childhood emotional maltreatment

Internal shame and psychological distress serially mediated the relationship between childhood emotional maltreatment (CEA, CEN) and binge eating. This finding is consistent with past childhood trauma literature which shows that childhood emotional maltreatment is a stronger predictor of adult depressive symptoms than other forms of maltreatment [43], and more recent research indicating that both CEA and CEN are linked to depressive symptoms via internal shame [61, 66]. Our results suggest that experiences of emotional maltreatment may be internalised, developing into longstanding beliefs about one’s inadequacy or worthlessness, which feeds psychological distress. Childhood emotional maltreatment is also a robust predictor of poor emotion regulation in adulthood [8]. Therefore, it is possible that the contribution of childhood emotional maltreatment to increased psychological distress, alongside poor emotion regulation skills, leads to the use of binge eating as a dysfunctional strategy to regulate distress [39].

Internal shame was found to contribute to body shame. This finding is consistent with previous research linking these forms of shame [46]. Internal shame might become associated with one’s body, in part due to the overvaluation of weight and shape in western and westernised cultures [35, 70]. Binge eating may then be used as a maladaptive strategy to experience temporary relief or attentional redirection from the experience of body shame [18].

### Childhood sexual abuse

The relationship found between CSA and binge eating is well supported by past literature [33]. However, to our knowledge, the present study is the first to identify body shame as a factor that fully mediates this relationship. Literature linking CSA to eating disorder pathology shows that there are stronger associations between CSA and bulimic pathology compared to restrictive eating behaviours [50]. It is theorised that binge-purge behaviour might serve as a way to regulate distress, to express anger, or as a physical manifestation of “cleansing” the body [62]. Our results add to this literature, indicating that non-compensatory binge eating may be used to alleviate body shame. Previous research shows that individuals who experience CSA may develop feelings of disgust or shame towards their own bodies [3]. It is possible that binge eating may be then used to modify one’s body to acquire and maintain a less attractive appearance and avoid future abuse, e.g., via weight gain [36], or to regulate body-related distress [20].

### Childhood physical maltreatment

Childhood physical maltreatment (CPA, CPN) was linked to binge eating via psychological distress. The association between childhood physical maltreatment and the development of adulthood depression and anxiety is well-established [40]. It is theorised that childhood physical maltreatment is a non-specific risk factor for psychopathology broadly in adulthood via a range of mediating mechanisms including low self-esteem [4] and insecure attachment [72]. Our results suggest that psychological distress also mediated this relationship, and that binge eating may be used to alleviate this distress.

### Overvaluation of shape and weight

Overvaluation of shape and weight mediated a feedback loop from binge eating to both internal shame and body shame. These findings were consistent with past research [34, 57], and suggest that the physical and psychological sequalae of binge eating (i.e., weight gain and increased overvaluation) further strengthen the shame-binge eating relationship. This finding highlights the central role of shame in perpetuating binge eating symptoms, and so the potential benefits in shame-focussed psychological treatment in BED.

### Limitations and future directions

This study has several limitations which must be considered in evaluating our results and conclusions. Considering that the data is cross-sectional, we are unable to infer causality among the variables. For instance, longitudinal research is required to test our hypothesis that body shame stemming from CSA may be reduced via binge eating. Our sample was non-clinical, and limited in diversity, with most participants being female, white, well-educated, and from a western country, therefore it is unclear whether our results generalise to the wider BED population. Accordingly, future research should attempt to replicate current findings using a clinical, representative sample. Another possible limitation was our use of dichotomous variables in our assessment of childhood maltreatment. While this measure shows strong concurrent validity with longer form measures of childhood trauma [54, 65], we were not able to examine severity levels of each type of childhood maltreatment which may have affected results.

### Conclusion and clinical implications

Our findings underscore the importance of screening for and treating the psychological effects of childhood maltreatment in BED presentations. Our results also highlight the importance of considering how the long-term consequences of childhood maltreatment may play an ongoing role in binge eating symptoms in adulthood, via increased internal shame, body shame, and/or psychological distress. Clinicians should screen for childhood maltreatment when treating individuals with BED. In presentations where childhood maltreatment experiences are reported, clinicians should also assess for and treat symptoms of internal shame, body shame, and psychological distress which may maintain binge eating. For individuals with high levels of internal shame and body shame, targeted interventions such as compassion focussed therapy for eating disorders (CFT-E) [31] should be considered. For those experiencing shame that is closely intertwined with specific childhood memories of abuse, interventions that target those shame memories such as eye movement desensitisation and reprocessing (EMDR) therapy may be indicated [67]. Gold standard treatments for BED such as enhanced cognitive behavioural therapy (CBT-E) [32] might also be adapted for shame-prone individuals with BED. For instance, targeting shame cognitions may be beneficial when using this treatment. Furthermore, for individuals with a history of CSA who experience body shame, a focus on treating body shame rather than body dissatisfaction may lead to more success in reducing binge eating. Future treatment development research for individuals with BED and a childhood maltreatment history could examine the efficacy of interventions such as CFT-E [31] that treat internal shame and body shame.

## Data Availability

The data that supports findings of this study are available, upon reasonable request, from Elyse O’Loghlen [elyseologhlen@swin.edu.au], for the duration of her employment at Swinburne University of Technology.
