# Correction: Childhood maltreatment, shame, psychological distress, and binge eating: testing a serial mediational model

**DOI:** 10.1186/s40337-023-00951-4

**Published:** 2023-12-19

**Authors:** Elyse O’Loghlen, Roslyn Galligan, Sharon Grant

**Affiliations:** Department of Psychological Sciences, Swinburne University of Technology, Hawthorn, VIC 3122 Australia

**Correction: Journal of Eating Disorders (2023) 11:96** 10.1186/s40337-023-00819-7

The original article [1] has incomplete funding information. The sponsorship from Takeda Pharmaceutical Australia to cover the Article Processing Charge was not mentioned in the Funding note.

The correct Funding note is as follows:

**Funding**

This work was supported by an Australian Government Research Training Program Scholarship. The article has received sponsorship from Takeda Pharmaceutical Australia to cover the Article Processing Charge.

The original article [1] has been corrected.
